# A clinicopathological study about the epidemiology of granulosa cell tumors in Lebanon

**DOI:** 10.1186/s12885-024-12047-6

**Published:** 2024-03-06

**Authors:** Sana Yaacoub, Labib Hajj, Aya Khairallah

**Affiliations:** Faculty of Medical Sciences, Lebanese University, Beirut, Lebanon

**Keywords:** Cord-sex tumor, Granulosa, Adult type, Juvenile type, Incidence, Lebanese population

## Abstract

**Background:**

Granulosa Cell Tumors (GCT) are considered the most frequent type of sex-cord stromal tumors. These tumors constitute 3-6% of neoplasms of the ovaries. GCTs are divided into 2 types: Juvenile GCT (JGCT) and Adult GCT (AGCT). Most patients are diagnosed early in the course of the disease and tend to have a favorable prognosis. In the surgical treatment of GCT, two main factors play role in the determination of feasibility of the surgery: age and tumor stage.

**Methods:**

A retrospective study was conducted on 65 consecutive female patients diagnosed with ovarian GCT at different hospitals across Lebanon who were referred to the National Institute of Pathology, Beirut-Lebanon, between January 2000 and January 2020. Then, they were divided according to types: adult versus juvenile type. Statistical analysis was carried out using Stata, version 16.

**Results:**

The incidence of GCT in a Lebanese population was 16.2 per million per year. The mean age of the studied population was 55.6 years. AGCT was the most common with a prevalence of 91% versus 19% for JGCT. Also, inhibine (the most important immunomarker) was found in 77.2% of adult cases. High mitotic index and high tumor size which are predictors for poor prognosis were respectively 20% and 36.9%. Concerning the histopathological features, Grooved nuclei and Exner bodies were less frequently observed in juvenile type (16.7% for both) compared to adult type (36.9%). Most patients with GCT were diagnosed in the early course of disease mainly due to the manifestation of the symptoms as abdominal pain, postmenopausal bleeding or intermenstrual bleeding, and the good diagnosis and screening practices in Lebanon. Regarding the recurrent cases, a significant correlation with high mitotic index (76.9%), high tumor size (92.3%) and advanced stage (46% for stage 3 and 46% for stage 4) was found with a p < 0.05.

**Conclusions:**

The incidence of GCT in the Lebanese population is 16.2 per million per year. The majority of patients with GCT in Lebanon are of Adult type representing around 90% of cases. Older age, high mitotic index and big tumor size are predictors for poor outcomes.

## Introduction

Granulosa Cell Tumors (GCTs) are the most commonly diagnosed sex cord-stromal tumors accounting for 5% of malignant ovarian tumors and 90% of sex cord-stromal tumors [1, 2]. About 21,000 women are diagnosed with ovarian tumors every year in the United States leading to the death of around 14,000 [3]. Of these women, 2% have GCT where almost 95% have the adult type (AGCT) and the remaining have the juvenile type (JGCT) [4]. As the name indicates, GCTs originate from the ovarian granulosa cells that represent the somatic component of the follicles of the ovary. Granulosa cells produce sex steroids and various growth factors which are mandatory for ovulation and folliculogenesis [5, 6].

GCTs have architectural patterns and may show a combination of macro-follicular, micro-follicular, trabecular, insular and cystic morphologies [7]. GCT patients clinically present with abdominal bloating, abdominal pain, fullness, pelvic mass, hormonal events (postmenopausal bleeding or intermenstrual bleeding) [8]. Other possible symptoms include nausea, dizziness, shoulder pain, and fullness [1]. Histologically, the architecture of JGCT is solid or multinodular with rare follicular differentiation, the cells are notched with more pronounced cellular pleomorphism and frequent luteinization, whereas the adult type has five different architectural forms: micro-follicular, macro-follicular, insular, trabecular, spindle-shaped or sarcomatoid. Cells of AGCT have pale nucleoli, ovoid shape, may have luteinization and a high aspect of notch in coffee-bean appearance. In addition, AGCT has Call Exner bodies, that are characteristic of this tumor, and a low mitotic index [9].

There are different stages of GCT. Stage 1, constituting 70% of all GCTs, is characterized by tumor growth in one ovary (unilateral). Stage 2 is defined by growth in either one or both ovaries (unilateral or bilateral) with presence of pelvic adhesions. Stage 3 is defined by growth in one or both ovaries (unilateral or bilateral) with peritoneal adhesions or positive nodes. Stage 4 is defined by growth in one or both ovaries (unilateral or bilateral) with distant metastasis (liver, bone…) [6, 10, 11].

The most important prognostic factor is staging [12]. Prognostic factors are divided into risk categories: low risk, intermediate risk and high risk [13]. The low risk is stage I categorized patients who have no need for post-op therapy with a survival rate of 90%. On the other hand, patients with high risk stage I or low risk stage II need adjuvant chemotherapy or radiotherapy. Finally, the high risk category is found in Stage III and IV requiring chemotherapy or whole abdominal radiotherapy [5, 10, 12]. Recurrences in patients with AGCTs tend to be later than in patients with JGCTs [14].

In the Middle East, and especially in Lebanon, there is a substantial lack of epidemiological data regarding GCT. Furthermore, the fork head box protein L2 (FOXL2) test which differentiates GCT from other cord sex tumors is not covered by a third payer. Accordingly, there is a dire need to estimate the incidence of GCT in the Lebanese population while investigating the variant types, histopathological features, predictors for recurrent cases and immunomarkers and comparing the situation to other developed countries. This would help in emphasizing the importance of awareness campaigns and the different tumor characteristics between different populations that may be explained by genetic variability.

The primary objective of this study was to determine the incidence of juvenile and adult types of GCT in Lebanon along with comparing them to other countries, assessing the number of GCT compared to all ovarian tumors, and determining the prognostic factors and the recurrence rate of GCT in Lebanon.

## Methods

### Study design and population

This study was an observational retrospective monocentric study conducted on patients referred to National Institute of Pathology, Beirut-Lebanon between January 2000 and January 2020.

A total of 65 female patients are included. Patients were eligible for participation in the study if they were diagnosed with ovarian GCT. All patients with benign tumors, different tumor types and incomplete data were excluded. The studied population was furthermore divided into 2 groups; AGCT and JGCT. Finally, the recurrent cases were investigated to detect the predictors for recurrence, bad prognosis.

### Procedures of data collection measurements

Tumor staging and patients’ data were collected from various hospitals and centers: Ain Wazein Hospital, Haroun Hospital, Dahr El Bachek Hospital, Middle East Institute of Health University Hospital (MEIH) Bsalim, American University Hospital (AUH), Centre Hospitalier du Nord (CHN), and Mazloum Hospital.

All pathology results performed between the defined study period (2000–2020) were analyzed. All pathology exams were done by the same reference physicians. Data concerning the demographic characteristics, diagnostic criteria, recurrence, positive immunomarkers (Calretinin, Cytokeratin, CD99 and Vimentin) and tumor characteristics like mitotic index, size, presence of grooved nuclei or exner bodies, macro- or micro-follicular, trabecular and insular aspects were collected.

### Data analysis

Quantitative data were summarized with means and standard deviations while qualitative data were summarized with count and percentage. The incidence was calculated according to the international defined formula in public health: number of new cases of disease during specified time interval/summed person-years of observation or average population during time interval. T student test was used for pairwise comparison of quantitative data whereas Chi-square test was used for pairwise comparison of qualitative data. A binary logistic regression was performed to investigate the independent variables correlated to recurrent cases, adult and juvenile tumors types. A p-value < 0.05 was considered significant. All statistical analysis was carried out by Stata, version 16.

## Results

### Patients’ characteristics and incidence of ovarian GCT

The mean age of the studied population was 55.6 years. Out of 65 patients: 6 (9.2%) had juvenile type, 59 (90.8%) had adult type, 24 (36.9%) had a high tumor size (> 10 cm), 27 (41.5%) had grooved nuclei, 25 (38.5%) had exner bodies, 8 (12.3%) were macrofollicular, 13 (20%) were microfollicular, 14 (21.5%) were trabecular, 7 (10.8%) were insular. The number of new cases during the study period was 65 and the summed person-years of observation was (3.5 millions + 4.5millions/2). The incidence of ovarian granulosa tumor in our studied population is 16.2 per million per year. Baseline characteristics are shown in Table 1.

**Table 1:** Characteristics of the studied population

Criteria	Whole Population (N = 65*)*	JGCT (N = 6)	AGCT (N = 59)
Mean age	55.6 years	21 years	60 years
Macrofollicular	8 (12.3%)	1 (16.7%)	7 (11.86%)
Microfollicular	13 (20%)	3 (50%)	10 (16.9%)
High mitotic index	13 (20%)	1 (16.7%)	12 (20.3%)
High tumor size	24 (36.9%)	4 (66.7%)	20 (33.9%)
Grooved nuclei	27 (41.5%)	1 (16.7%)	26 (40%)
Exner bodies	25 (38.5%)	1 (16.7%)	24 (36.9%)
Trabecular	14 (21.5%)	1 (16.7%)	13 (22.03%)
Insular	7 (10.8%)	1 (16.7%)	6 (10.17%)
Recurrence	13 (20%)	1 (16.7%)	12 (20.3%)

JGCT: Juvenile Granulosa Cell Tumor, AGCT: Adult Granulosa Cell Tumor

### JGCT versus AGCT

Taking into consideration the juvenile group (*N* = 6), the mean age was 21 years. Out of 6 patients: 1 (16.7%) had recurrent disease. Regarding tumor characteristics: 1 (16.7%) had high mitotic index (defined as greater than 4–10/10Hpf), 4 (66.7%) had a high tumor size (> 10 cm), 1 (16.7%) had grooved nuclei, 1 (16.7%) had exner bodies, 1 (16.7%) were macrofollicular, 3 (50%) were microfollicular, 1 (16.7%) were trabecular, 1 (16.7%) were insular. Statistical analysis shows a significant correlation between juvenile ovarian GCT type and young age, and high tumor size (p-value < 0.05*) (Table 1). Young patients (age less than 30 years old) are more predisposed to ovarian GCT, also those with high tumor size (more than 10 cm) are more exposed to develop GCT than others

Taking into consideration the adult group (*N* = 59), the mean age was 60 years. Out of 59 patients: 12 (20.3%) had recurrent disease. Regarding tumor characteristics: 12 (20.3%) had high mitotic index (defined as greater than 4–10/10Hpf), 20 (33.9%) had a high tumor size (> 10 cm), 26 (40%) had grooved nuclei, 24 (36.9%) had exner bodies, 7 (11.86%) were macrofollicular, 10 (16.9%) were microfollicular, 13 (22.03%) were trabecular, 6 (9.2%) were insular. Statistical analysis shows a significant correlation between adult ovarian GCT type and older age (> 50y) (*p* < 0.05) (Table 1)

### Recurrent GCT cases

Regarding recurrence while most cases were observed in adult type (92.3 vs. 7.7%), statistical analysis didn’t show a significant relationship with p-value = 0.09. The mean age of recurrent cases was 61.7 years. In recurrent cases, high mitotic index was observed in 76.9% (10 cases), high tumor size in 92.3% (12 cases), grooved nuclei in 30.7% (4 cases) and exner bodies in 38.4% (5 cases). 15.4% of recurrent cases were macrofollicular while 23% were microfollicular, 15.4% were trabecular and 7.7% were insular. Statistical analysis showed a significant relationship between recurrence and the presence of high mitotic index and high tumor size (Table 2) (p-value = 0.09)

**Table 2:** Characteristics of patients with recurrent disease (N = 13)

Mean age	61.7 years
Adult type	12 (92.3%)
Juvenile type	1 (7.7%)
High mitotic index	10 (76.9%)
High tumor size	12 (92.3%)
Grooved nuclei	4 (30.7%)
Exner bodies	5 (38.4%)
Macrofollicular	2 (15.40%)
Microfollicular	3 (23%)
Trabecular	2 (15.40%)
Insular	1 (7.70%)
Stage 1	0 (0%)
Stage 2	1 (7.7%)
Stage 3	6 (46%)
Stage 4	6 (46%)

View the loss to follow-up, we have defined the stage of 35 patients. Indeed, 7 were classified in stage 4 (23.3%), 9 in stage 3 (25.7%), 12 (34.3%) in stage 2 and 10 in stage 1 (28.6%). Regarding the tumor types: 5 out of 7 in stage 4, 4 out of 9 in stage 3, 7 out of 12 in stage 2 were adult type

All recurrent cases were stages 3 and 4 except for 1 which was stage 2. A significant relationship after using Mann-Whitney U test to study the difference of prevalence of recurrence between high stage group (3 and 4) versus low stage group (1 and 2)

### Immunomarkers

Immunomarkers were positive for inhibine in 75%, for calretinin in 78.3%, for cytokeratin in 50%, for CD99 in 82.6% and for vimentin in 100%. The distribution among adults and juvenile and the incidence of recurrence among each type are detailed below (Table 3)

**Table 3:** Incidence and distribution of immunomarkers along with comparative percentages

Immuno-marker	Number of performed tests	Positive			Negative		
		Count	AGCT	JGCT	Count	AGCT	JGCT
Inhibine	40	30 (75%)	27 (90%)	3 (10%)	10 (25%)	8 (80%)	2 (20%)
Calretinin	23	18 (78.3%)	16 (88.9%)	2 (11.1%)	5 (21.7%)	5 (100%)	0 (0%)
Cytokeratin	20	10 (50%)	9 (90%)	1 (10%)	10 (50%)	9 (90%)	1 (1%)
CD99	23	19 (82.6%)	17 (89.5%)	2 (10.5%)	4 (17.4%)	4 (100%)	0 (0%)
Vimentin	18	18 (100%)	16 (88.9%)	2 (11.1%)	0 (0%)	0 (0%)	0 (0%)

JGCT: Juvenile Granulosa Cell Tumor, AGCT: Adult Granulosa Cell Tumor

## Discussion

Our study showed a global incidence rate of ovarian GCT in Lebanese population around 16.2 per million per year. Based on the fact that National Institute of Pathology received tests from different hospitals of several Lebanese regions, we can consider that this population sample reflected the status of general Lebanese population. The incidence was calculated by using the defined formula according to the rules in epidemiology of public health which was the number of new cases of disease during specified time interval/summed person-years of observation or average population during time interval. The new cases between this period were 65 cases and the summed person-years of observation was (3.5 million + 4.5 million/2). The estimated exposed Lebanese population is 3.5 million in 2000 and 4.5 million in 2020. So overall, the summed person-years is 4 million. The adult tumor type was more common than juvenile type and was observed more in older individuals. Also, the recurrent cases were more often observed in adult type without signaling a significant correlation (*p* = 0.09). An older age, high mitotic index and high tumor size are identified as predictors for high risk of recurrence. All these resumed the main findings of our study

Based on the world cancer report, the cases of ovarian cancer increased annually by 239,000 accounting 10,000 for the adult GCT. The definite incidence of ovarian GCT isn’t known but it varies between 5 and 14 per million per year in the European countries such as Denmark, Sweden, Finland and Netherlands and it is estimated at 9.9 per million in the United States. Also, Brik et al. showed that the international incidence of GCT ranges between 4.7 to 16 per million [15]. Subsequently, our calculated incidence seems on the upper limit while the distribution between Juvenile (9.2%) and Adult type accounting for 90.8% of all cases is closer to the global norms (95% for adult versus 3–5% for juvenile). This higher incidence may be explained by the low rate of hysterectomy and oral contraceptive use in the Lebanese population knowing that these factors on top of tubal ligation are known to have been reduced the risk of ovarian tumor. Furthermore, the lack of doing the FOXL2 test which confirms the case of GCTs or differentiates it from other tumors is a reason supporting the hypothesis of an over-diagnosis

The mean age was 13 in the juvenile group and 54.5 in the adult group. In parallel, a higher mean age was reported in the adult group (60 years old) in European population compared to the juvenile group (21 years old) [16]. The lower mean age of ovarian cancer in our studied population could reflect the effectiveness of awareness campaigns and screening programs as the important compliance of the Lebanese population. However, the younger mean age of juvenile tumor may reflect a greater impact of the hereditary risk factor.

Considering the histopathological features, GCT was respectively 20% trabecular, 15.4% microfollicular, 10.7% macrofollicular and 9.2% insular while in a studied European population the percentage was respectively 11%, 22%, 6% and 14%. In parallel, inhibine was positive in 75%, calretinin 78.3% and CD99 in 82.6%. However, previous study performed in an European population revealed a positive inhibine in 100% of AGCTs and for calretinin [16, 17]. The significant difference in these findings may be due to the advanced techniques, more available tests, and role of genetic component. Also, in our small sample we highlight a significant higher rate of positive inhibine in the adult group (90% vs. 80%). Moreover, previous studies have shown that all AGCT and JGCT presented hypercellularity, clusters, sheets, single cells and naked nuclei whereas exner bodies and grooves were seen only in AGCTs. In opposition, JGCT showed more striking cellular atypia. These findings were also highlighted in our results where Call Exner bodies were almost shown in adult type except for only one case of juvenile type. Moreover, the difference in the immunohisto pathological features may be due to genetic factor variations between the Lebanese and European population and also it may be explained by the presence of numerous cases classified by default as GCTs view the non-feasibility of FOXL2 test

AGCTs are known for their relatively good prognosis and tendency for late relapse. In recent studies, the rate of tumor recurrence varies widely from 5–64%. Indeed, the rate of recurrence was 20% in our studied population and it was significantly more observed in the highest stage group (3 and 4) *p* < 0.05. Several predictor factors were identified like tumor size, stage, histological features such as mitotic index and the residual tumor. Also, our study emphasized that big tumor size (> 10 cm), high mitotic index, advanced age and stage were determinants predictor for recurrence. It is noteworthy that most patients with GCT are diagnosed in the early course of the disease due to the severity of the symptoms associated with GCT. Most women face abdominal pain along with menstrual abnormality or bleeding even after menopause, thus driving them to seek clinical examination and histopathologic analysis. Even more, this indicates that awareness campaigns and screening programs are effective in Lebanon. Tumor stage is the only clinical prognostic factor that is reported positive in virtually all studies and also in multivariate analyses. Larger tumor size has been associated with poorer outcome also in multivariate analysis. However, this is not a consistent finding in other series. Ud Din et al. reviewed 156 cases and concluded that tumor size is associated with higher risk of recurrence [18]. Preoperative or preoperative tumor rupture has been proposed to influence particularly the risk for tumor recurrence. Histological prognostic factors include mitotic index, poor differentiation of the tumor, and nuclear atypia. Although, cellular atypia and mitotic index may help in determining the prognosis of GCT, they are difficult to evaluate. Like us, numerous studies have found a correlation between higher mitotic index and poor prognosis of GCT. These results may be due to similar common characteristics between this studied population compared to others or may confirm the universal features of recurrent cases of GCT [15–17]. Although FOXL2 mutation is associated with early relapse and worse outcome [19], other alternative immune stains, such as CD56, SMAD3, Inhibin A and Calretinin, can be utilized as independent predictors of GCT recurrence [20]

### Study limitations

Considering the fact that GCTs can be associated in approximately 10% of the cases with Endometrial Carcinoma, we strictly reduced our study population to patients who were free of this pathology or other tumors. Further limitations of our retrospective study were the loss of data of many patients from hospital directories, the death of many patients with inability for file recovery from the hospitals and the small number of cases in Lebanon

### Study perspectives

This study aims to evaluate the incidence of ovarian GCT in a Lebanese population and to compare the findings to these found in other population like European and US population. Moreover, we aim to highlight on the impact of these tumors and the importance to improve the diagnostic method. Especially, we emphasize on the interest to perform the FOXL2 test which help to differentiate the several types of sex-cord tumors

Indeed, we aim to focus on the interest for the community to provide a third part payer for this test. Furthermore, view that the calculated incidence is in the upper limit of the international range; a survey study aiming to screen the using of oral contraceptive pills, gynecologists practice, awareness campaign is useful. More developed randomized clinical trials investigating the management of GCTs in Mediterranean population and the genetic variation are recommended. Further, future epidemiological studies involving the Lebanese population is needed, especially view the lacking of similar data in our community where the most data are extrapolated from the European countries

Lastly, early stage is associated to better prognosis. Also, early detection or early diagnosis leads to a better outcome. All these result in the importance of population awareness. A message that we hope to spread it for our Lebanese community by showing the highest incidence of this tumor type. Moreover, the cost of FOXL2 test must be estimated and economic facilities will be planified in order to perform more commonly this useful diagnostic test

## Conclusion

In conclusion, GCTs have a favorable outcome because the majority of patients are diagnosed in early stages. GCT are uncommon ovarian neoplasms in Lebanon and abroad (i.e. US) with the majority being of Adult type. Patients younger than 30 years old are more predisposed to ovarian GCT, and those with tumor size greater than 10 cm are more predisposed to GCT development than others. Furthermore, diagnostic criteria are mandatory to differentiate between JGCT and AGCT. It’s also important to note that GCT patients may relapse even 1 year after initial treatment which is why follow-up with tumor markers is necessary and possibly life-saving!

Note that many prognostic factors have been identified to lead to a poorer outcome and to a higher recurrence rate: a more advanced tumor stage, classified as the most important, a higher mitotic index, a larger tumor size, an advanced age at the time of diagnosis and the presence of a residual tumor after surgery

Lastly, the Lebanese situation of GCT is close to what’s reported in the literature in different countries and variant population. Genetic variability could be the potent responsible agent explaining the difference in histopathological features

## Data Availability

The datasets analyzed during the current study are available in the Zenodo repository, [data will be uploaded and web link will be shared upon accepting the paper for publication]

## Abbreviations

AGCT: Adult Granulosa Cell Tumor
AUH: American University Hospital
CHN: Centre Hospitalier du Nord
FOXL2: Fork Head Box Protein L2
GCT: Granulosa Cell Tumor
Hpf: High-Power Fields
JGCT: Juvenile Granulosa Cell Tumor
MEIH: Middle East Institute of Health University Hospital Research Terms and Definitions
Adult Granulosa Cell Tumor: It refers to GCT in females older than 50 years of age
Juvenile Granulosa Cell Tumor: It refers to GCT in females younger than 30 years of age
Tumor size: It is defined as the maximum diameter of the lesion as inspected in pathologic examination. A diameter greater than 10 cm refers to a high tumor size; a diameter less than 10 cm refers to a low tumor size
Mitotic index: It refers to the number of mitoses in 10 consecutive high-power fields (Hpf) from the lesion. When it is greater than 4-10/10Hpf, it reflects high mitotic index; when it is lower than this value, it reflects a low mitotic index

## References

[1] Dridi M, Chraiet N, Batti R, Ayadi M, Mokrani A, Meddeb K, Yahiaoui Y, Raies H, Mezlini A. Granulosa Cell Tumor of the Ovary: A Retrospective Study of 31 Cases and a Review of the Literature. Int J SurgOncol. 2018;2018:4547892. 10.1155/2018/4547892. PMID: 29796312. https://doi.org/10.1155/2018/4547892. 10.1155/2018/4547892 PMC5896205 29796312

[2] Shah SP, Köbel M, Senz J, Morin RD, Clarke BA, Wiegand KC et al. Mutation of FOXL2 in granulosa-cell tumors of the ovary. N Engl J Med. 2009;360(26):2719-29. 10.1056/NEJMoa0902542. PMID: 19516027. https://www.nejm.org/doi/full/10.1056/NEJMoa0902542. 10.1056/NEJMoa0902542 19516027

[3] Timmons J. (2004, November 15). Ovarian Cancer by the Numbers. Retrieved July 15, 2020, from https://www.healthline.com/health/cancer/ovarian-cancer-facts-statistics-infographic.

[4] Michener C. Granulosa-theca cell tumors. Medscape Ref. 2010. http://emedicine.medscape.com/article/254489.

[5] Jamieson S, Fuller PJ. Molecular pathogenesis of granulosa cell tumors of the ovary. Endocr Rev. 2012;33(1):109– 44. 10.1210/er.2011-0014. PMID: 22240241. https://doi.org/10.1210/er.2011-0014. 10.1210/er.2011-0014 22240241

[6] Schumer ST, Cannistra SA. Granulosa cell tumor of the ovary. J Clin Oncol. 2003;21(6):1180-9. 10.1200/JCO.2003.10.019. PMID: 12637488. https://ascopubs.org/doi/10.1200/JCO.2003.10.019. 10.1200/JCO.2003.10.019 12637488

[7] Guleria P, Kumar L, Kumar S, Bhatla N, Ray R, Singhal S (2020). A clinicopathological study of granulosa cell tumors of the ovary: can morphology predict prognosis?. Indian J Pathol Microbiol.

[8] Yang AD, Curtin J, Muggia F (2018). Ovarian adult-type granulosa cell tumor: focusing on endocrine-based therapies. Int J EndocrOncol.

[9] Shamsudeen S, Mahdy H. Granulosa Theca Cell Cancer. [Updated 2022 Dec 11]. In: StatPearls [Internet]. Treasure Island (FL): StatPearls Publishing; 2023 Jan-. Available from: https://www.ncbi.nlm.nih.gov/books/NBK565872/.

[10] Khosla D, Dimri K, Pandey AK, Mahajan R, Trehan R. Ovarian granulosa cell tumor: clinical features, treatment, outcome, and prognostic factors. N Am J Med Sci. 2014;6(3):133-8. 10.4103/1947-2714.128475. PMID: 24741552. http://www.najms.org/text.asp?2014/6/3/133/128475. 10.4103/1947-2714.128475 PMC3978936 24741552

[11] Björkholm E, Silfverswärd C. Prognostic factors in granulosa-cell tumors. GynecolOncol. 1981;11(3):261– 74. 10.1016/0090-8258(81)90040-8. PMID: 7250754. 10.1016/0090-8258(81)90040-8 7250754

[12] Miller BE, Barron BA, Wan JY, Delmore JE, Silva EG, Gershenson DM. Prognostic factors in adult granulosa cell tumor of the ovary. Cancer. 1997;79(10):1951-5. PMID: 9149022. https://acsjournals.onlinelibrary.wiley.com/doi/10.1002/(SICI)1097-0142(19970515)79:10%3C1951::AID-CNCR16%3E3.0.CO;2-U 10.1002/(sici)1097-0142(19970515)79:10<1951::aid-cncr16>3.0.co;2-u 9149022

[13] Fox H, Agrawal K, Langley FA. A clinicopathologic study of 92 cases of granulosa cell tumor of the ovary with special reference to the factors influencing prognosis. Cancer. 1975;35(1):231– 41. PMID: 1109770. https://acsjournals.onlinelibrary.wiley.com/doi/10.1002/1097-0142(197501)35:1%3C231::AID-CNCR2820350128%3E3.0.CO;2-O 10.1002/1097-0142(197501)35:1<231::aid-cncr2820350128>3.0.co;2-o 1109770

[14] Kattan J, Droz JP, Culine S, Duvillard P, Thiellet A, Peillon C. The growing teratoma syndrome: a woman with nonseminomatous germ cell tumor of the ovary. GynecolOncol. 1993;49(3):395-9. 10.1006/gyno.1993.1147. PMID: 8314544. https://doi.org/10.1006/gyno.1993.1147. 10.1006/gyno.1993.1147 8314544

[15] Bryk S. Epidemiological, clinical, and prognostic factors in adult-type ovarian granulosa cell tumors. Helsinki: Univ Helsinki, 2017. 119 p. https://nfog.org/wp-content/uploads/2018/01/EPIDEMIOLOGICAL-CLINICAL-AND-PROGNOSTIC-FACTORS-IN-ADULT-TYPE-OVARIAN-GRANULOSA-CELL-TUMORS.pdf.

[16] Babarović E, Franin I, Klarić M, Ferrari AM, Karnjuš-Begonja R, Eminović S et al. Adult granulosa cell tumors of the ovary: a retrospective study of 36 FIGO stage I cases with emphasis on prognostic pathohistological features. Anal Cell Pathol (Amst). 2018;2018:9148124. 10.1155/2018/9148124. PMID: 30186737. https://doi.org/10.1155/2018/9148124. 10.1155/2018/9148124 PMC6116457 30186737

[17] Bryk S, Pukkala E, Martinsen JI, Unkila-Kallio L, Tryggvadottir L, Sparén P et al. Incidence and occupational variation of ovarian granulosa cell tumours in Finland, Iceland, Norway and Sweden during 1953–2012: a longitudinal cohort study. BJOG. 2017;124(1):143-9. 10.1111/1471-0528.13949. PMID: 26924812. https://doi.org/10.1111/1471-0528.13949. 10.1111/1471-0528.13949 26924812

[18] Ud Din N, Kayani N. Recurrence of adult granulosa cell tumor of the ovary: experience at a tertiary care center. Ann Diagn Pathol. 2014;18(3):125-8. 10.1016/j.anndiagpath.2014.02.002. PMID: 24630126. https://doi.org/10.1016/j.anndiagpath.2014.02.002. 10.1016/j.anndiagpath.2014.02.002 24630126

[19] Kraus F, Dremaux J, Altakfi W, Goux M, Pontois L, Sevestre H et al. FOXL2 homozygous genotype and chromosome instability are associated with recurrence in adult granulosa cell tumors of the ovary. Oncotarget. 2020;11(4):419–428. 10.18632/oncotarget.27447. PMID: 32064045. https://pubmed.ncbi.nlm.nih.gov/32064045/. 10.18632/oncotarget.27447 PMC6996913 32064045

[20] Sakr S, Abdulfatah E, Thomas S, Al-Wahab Z, Beydoun R, Morris R et al. Granulosa Cell Tumors: Novel Predictors of Recurrence in Early-stage Patients. Int J Gynecol Pathol. 2017;36(3):240–252. 10.1097/PGP.0000000000000325. PMID: 28727617. https://pubmed.ncbi.nlm.nih.gov/28727617/. 10.1097/PGP.0000000000000325 PMC6171102 28727617

